# Interpreting the risk of postoperative post-neuraxial puncture-site pain after cesarean section: a machine learning approach evaluating nalbuphine infiltration along the epidural puncture tract in a prospective observational cohort

**DOI:** 10.3389/fmed.2026.1908970

**Published:** 2026-07-29

**Authors:** Tianping He, Qiying Yan, Yan Yang, Qiuhao Shui, Jianfeng Huang, Mianhui Wang

**Affiliations:** 1 Department of Anesthesiology, Luxian People's Hospital, Luzhou, China; 2 Department of Pathology, Luxian People's Hospital, Luzhou, China; 3 Department of Anesthesiology, Luxian Hospital of TCM, Luzhou, China

**Keywords:** cesarean section, enhanced recovery after surgery, machine learning, nalbuphine, post-neuraxial puncture-site pain

## Abstract

**Background:**

Postoperative post-neuraxial puncture-site pain following combined spinal-epidural anesthesia can impede early maternal recovery.

**Methods:**

In this prospective observational cohort study (*n* = 473), parturients undergoing cesarean section were categorized into Group L (ropivacaine infiltration) or Group NR (nalbuphine–ropivacaine infiltration along the epidural tract). We evaluated early clinical outcomes and developed machine learning models to predict post-neuraxial puncture-site pain risk, utilizing SHapley Additive exPlanations (SHAP) to interpret feature associations.

**Results:**

Group NR exhibited lower incidences of acute post-neuraxial puncture-site pain, reduced opioid consumption, and attenuated early postpartum depressive symptoms compared to Group L (all *P* < 0.05). Among the predictive models, XGBoost demonstrated superior discriminative performance (AUC = 0.912). SHAP analysis indicated that Nalbuphine infiltration, body mass index, and maternal age were the primary contributors associated with the predicted risk, revealing non-linear dependencies.

**Conclusion:**

Local epidural infiltration of nalbuphine and ropivacaine may decrease acute post-neuraxial puncture-site pain incidence and improve early postpartum recovery. The XGBoost model, combined with SHAP analysis, suggests robust capabilities in predicting post-neuraxial puncture-site pain risk and elucidating complex clinical associations.

## 1 Introduction

Cesarean delivery is very common and combined spinal/epidural anesthesia is the preferred anesthesia method in obstetrics worldwide (1, 2). However, localized post-neuraxial puncture-site pain represents a highly prevalent and distressing complication of neuraxial blockade, hypothesized to be triggered by localized tissue trauma from the large-bore Tuohy needle (3, 4). Accumulating evidence indicates that this acute localized back pain substantially restricts early out-of-bed mobilization and impairs neonatal care during the critical early puerperium (5). Furthermore, clinical studies have suggested that severe acute pain in the early postpartum phase is strongly associated with elevated risks of maternal psychological distress, specifically acute postpartum anxiety and depressive symptoms (6, 7). Consequently, mitigating puncture-site trauma to minimize localized back pain has emerged as a key perioperative target under Enhanced Recovery After Surgery (ERAS) protocols in obstetrics.

Combined or multimodal approaches to providing pain relief are currently recommended to decrease the use of opioids systemically (8, 9). Nalbuphine is a mixed kappa-opioid receptor agonist/mu-opioid receptor antagonist drug that has shown promise in regional anesthesia with a good side-effect profile (10). The infiltration of anesthetics in the surgical incision is commonplace but the clinical consequences of infiltrating a mixture of nalbuphine and ropivacaine through the epidural puncture tract have not been studied (11). This targeted intervention is theorized to reduce localized tissue trauma and subsequent inflammatory pain response following puncture (12).

Besides novel analgesic interventions evaluated, it is important to identify parturients at high risk for post-neuraxial puncture-site pain in the postoperative period in order to provide individualized care (13). Predictive analyses in this field are usually carried out using logistic regression and may not be able to include complex combinations of interactions between the variables, such as body mass index, maternal age and procedural difficulty, that may be potentially non-linear (14). An alternative approach for modeling these multifaceted datasets is with the use of machine learning algorithms (15, 16). Additionally, by using SHapley Additive exPlanations (SHAP) framework, complex algorithmic outputs can be translated into clinically interpretable feature associations (17).

Hence, the objective of this prospective observational cohort study is to evaluate the relationship between local administration of a nalbuphine-ropivacaine mixture and the development of persistent post-neuraxial puncture-site pain in the early postpartum period and acute postoperative puncture-site pain. Furthermore, we want to create predictive models with different machine learning approaches and analyze them with SHAP to better understand the underlying non-linear relationships between features and help optimize obstetric pain management.

## 2 Materials and methods

### 2.1 Study design and ethical approval

The study was a single-center, prospective, observational cohort study, which was approved by the Ethics Committee of Luxian People's Hospital [Approval No. (Ethics) 2024 (Research) No. 006]. The study was carried out in compliance with the ethical principles of the 1975 Declaration of Helsinki (revised in 2013). Because this study was prospective and observational, utilizing de-identified clinical data collected during routine clinical practice with minimal risk to patients, the requirement for written informed consent was formally waived by the Ethics Committee of Luxian People's Hospital. Additionally, as this study evaluated established clinical protocols without investigator-assigned randomization or active interventional allocation, clinical trial registration was considered not applicable under institutional guidelines. The prospective enrolment and follow-up were carried out from July 2024 to January 2026.

### 2.2 Patient selection

Inclusion criteria were: primiparous women with full-term pregnancies (20–42 years old); American Society of Anesthesiologists (ASA) physical status II or III; undergoing elective cesarean section under combined spinal-epidural anesthesia. Patients were excluded if they had any of the following: (1) a history of chronic post-neuraxial puncture-site pain or other chronic pain syndromes before pregnancy, or active post-neuraxial puncture-site pain and pelvic girdle pain during pregnancy; (2) a psychiatric disorder (major depression or anxiety disorder); (3) contraindications to neuraxial anesthesia; (4) surgery under general anesthesia; or (5) incomplete key medical records.

### 2.3 Clinical variables and intervention

Patients were divided according to the method of intervention that was written in the epidural puncture procedure: Group L received local layered infiltration along the puncture tract with 4 ml of 0.5% ropivacaine (20 mg), whereas Group NR received a mixture of 10 mg nalbuphine and 20 mg ropivacaine, formulated to a total volume of 4 ml. The choice of intervention was determined strictly by clinician preference and departmental protocols, rather than specific patient characteristics. Prior to the withdrawal of the 17G Tuohy needle, the anesthetic mixture was infiltrated layer-by-layer (from the ligamentum flavum, interspinous ligament, supraspinous ligament, down to the subcutaneous tissue and skin) at a depth corresponding to the needle insertion (approximately 4–6 cm). Crucially, no medication from this infiltration was administered into the epidural space. Baseline predictors were carefully distinguished from postoperative outcomes, to avoid leakage in the model construction. The following ten baseline and procedural variables were identified as predictors: maternal age, body mass index, gestational age, weight gain in pregnancy, number of puncture attempts, anesthesia operation time, surgery time, blood loss during surgery, birth weight of the neonate and baseline maternal blood pressure. Clinical outcomes evaluated included the occurrence of acute postoperative post-neuraxial puncture-site pain on day 1. To capture provoked inflammatory hyperalgesia specifically resulting from needle-induced tissue trauma, this outcome was strictly defined as any localized tenderness over the epidural puncture tract (L2-3 or L3-4 interspace) upon light digital palpation. The assessment was standardized and performed at exactly 24 h postoperatively by an independent nurse blinded to group allocation, using a Visual Analog Scale [VAS 0–10, with VAS ≥ 1 indicating a positive sign of local tissue injury]. This strict anatomical localization was essential to distinguish puncture-site pain from generalized incisional pain or uterine cramping. Other evaluated outcomes included psychological parameters (SAS anxiety scores and EPDS depression scores), total sufentanil consumption at 24 h, number of effective PCIA presses and recovery indicators (time to first out of bed mobilization and time to first flatus).

### 2.4 Machine learning algorithms and model development

Four supervised machine-learning algorithms: Logistic Regression (LR), Support Vector Machine (SVM), Random Forest (RF), and eXtreme Gradient Boosting (XGBoost) were developed. Because the data were derived from an observational cohort, the inclusion of the intervention strategy as a predictor was not intended to estimate counterfactual individual treatment benefits. Instead, the model serves strictly as an analytical framework to identify and visualize complex, non-linear statistical associations between clinical features and the outcome. To maximize data utilization and ensure robust evaluation, internal validation was performed across the entire cohort (*n* = 473) using a strict 5-fold cross-validation workflow, rather than a single train-test split. Hyperparameters in the model were optimized by 5-fold cross-validation in the training set. For example, the XGBoost model parameters were: maximum tree depth of 4, learning rate (eta) was 0.05, subsample ratio was 0.8, and boosting rounds were 150. The class distribution was first checked for possible imbalance in the class distribution of the dataset. To ensure unbiased learning, algorithmic adjustments, such as class weights (e.g., the scale_pos_weight parameter in XGBoost) and stratified cross-validation were used. To strictly prevent information leakage, all data preprocessing, LASSO regularization, and recursive feature elimination were executed entirely within each respective training fold of the 5-fold cross-validation. All reported final model performance metrics (e.g., confusion matrix, accuracy, and AUC) were subsequently derived from the aggregated out-of-fold predictions. Moreover, global and local feature associations were interpreted using SHapley Additive exPlanations (SHAP) framework.

### 2.5 Statistical analysis

Baseline characteristics and clinical outcomes were compared by standard statistical analyses. The Shapiro-Wilk test was used to test for normal distribution of the continuous variables. Normally-distributed data were presented as mean ± standard deviation and compared using the independent-samples *t*-test, while non-normally distributed continuous variables were presented as median [interquartile range] and compared using the Mann–Whitney *U* test. Data for categorical variables were presented as frequency (percentages) and analyzed by the Chi-square test or Fisher's exact test as appropriate. The area under the receiver operating characteristic curve (AUC) was used to assess the model discriminative performance and DeLong test was used to compare differences in AUC values. Precision-Recall curves were also plotted to further measure precision with respect to class distribution. To control for potential selection bias and baseline confounding factors in this observational cohort, multivariable logistic and linear regression models were performed for single-point binary and continuous outcomes, respectively, to calculate adjusted odds ratios (aORs) or adjusted beta coefficients (β) after adjusting for maternal age, body mass index, gestational age, weight gain during pregnancy, and the number of puncture attempts. Furthermore, for longitudinal repeated-measures secondary outcomes (including SAS, EPDS, and QoR-15 scores evaluated across multiple time points), Generalized Estimating Equations (GEE) with an exchangeable correlation structure were constructed to model the longitudinal trajectories, integrating group, time, and group-by-time interaction terms to estimate adjusted effects over time, with the GEE-adjusted marginal means and 95% confidence intervals plotted as Supplementary Figure S1. Cumulative incidence curves using the Kaplan–Meier method were used to analyze time-to-event recovery outcomes with group comparison using the Log-rank test. A two-sided *P*-value < 0.05 was considered to indicate statistical significance. The analysis was done in R software.

## 3 Results

### 3.1 Baseline characteristics and feature selection

The total number of parturients in this study was 473: 207 in Group L and 266 in Group NR. Demographic and obstetric parameters such as maternal age, BMI and gestational age were well matched between the two groups (all *P* > 0.05, Table 1). Likewise, surgical and anesthesia-related factors, such as the number of multiple puncture attempts and the length of surgery were not statistically different. These results suggest that the cohort was homogenous at baseline and a good base on which to build further predictive modeling. It is worth mentioning that postoperative variables (pain scores and analgesic consumption) were not included in the feature set, as this would contaminate the data set.

**Table 1:** Baseline demographic and perioperative characteristics of the study population.

Variables	Total (*n* = 473)	Group L (*n* = 207)	Group NR (*n* = 266)	*P* Value
Demographics and obstetric data				
Age (years), Mean ± SD	29.4 ± 4.4	29.5 ± 4.3	29.3 ± 4.4	0.683
Body mass index (kg/m^2^), Mean ± SD	26.4 ± 3.6	26.5 ± 3.5	26.4 ± 3.7	0.834
Weight gain (kg), Mean ± SD	14.9 ± 3.9	14.9 ± 3.8	15.0 ± 3.9	0.673
Gestational age (weeks), Median [IQR]	39.0 [39.0–39.0]	39.0 [39.0–39.0]	39.0 [39.0–39.0]	0.704
Baseline SBP (mmHg), Mean ± SD	115.2 ± 8.9	115.6 ± 8.8	114.9 ± 9.0	0.428
Surgical and anesthesia data				
Puncture attempts > 1, *n* (%)	148 (31.3%)	59 (28.5%)	89 (33.5%)	0.292
Anesthesia operation time (min), Median [IQR]	5.0 [3.0–6.0]	5.0 [4.0–6.0]	5.0 [3.0–6.0]	0.928
Duration of surgery (min), Mean ± SD	47.8 ± 7.1	47.8 ± 7.3	47.9 ± 7.0	0.816
Intraoperative blood loss (ml), Mean ± SD	358.8 ± 50.3	357.4 ± 50.7	360.0 ± 50.0	0.583
Neonatal birth weight (g), Mean ± SD	3,241.5 ± 350.9	3,227.4 ± 345.5	3,252.5 ± 355.3	0.440
Postoperative outcomes (for reference)				
Total sufentanil at 24 h (μg), Mean ± SD	58.8 ± 4.3	61.2 ± 3.8	56.9 ± 3.7	**< 0.001**
PCIA effective presses at 24 h (times), Median [IQR]	3.0 [1.0–4.0]	4.0 [2.0–5.0]	1.0 [0.0–3.0]	**< 0.001**
Incidence of Acute post-neuraxial puncture-site pain at Day 1, *n* (%)	245 (51.8%)	150 (72.5%)	95 (35.7%)	**< 0.001**

Continuous variables with normal distributions are presented as Mean ± Standard Deviation (SD) and compared using the independent-samples *t*-test. Non-normally distributed variables are presented as Median [Interquartile Range, IQR] and compared using the Mann-Whitney U test. Categorical variables are presented as frequencies (percentages) and analyzed using the Chi-square test or Fisher's exact test, as appropriate.

**Figure 1:**
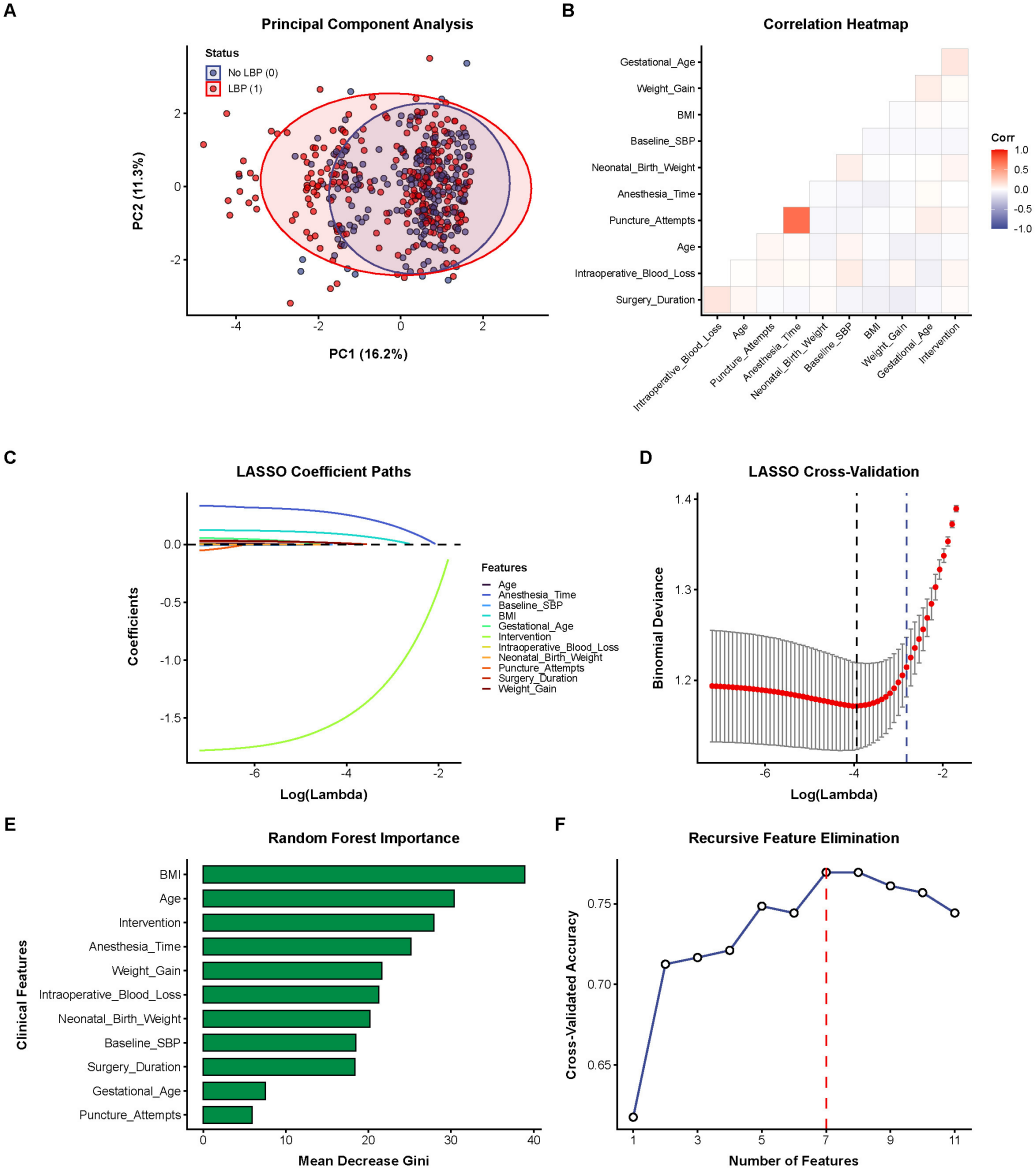
Feature selection and dimensionality reduction. **(A)** Principal Component Analysis (PCA) plot of the dataset. **(B)** Correlation heatmap of the included clinical variables. **(C)** LASSO regression coefficient paths. **(D)** LASSO cross-validation curve. **(E)** Feature importance ranking generated by the Random Forest model. **(F)** Recursive feature elimination (RFE) curve indicating the relationship between the number of features and cross-validated accuracy.

Principal Component Analysis was used to investigate the spatial distribution of the data set (Figure 1A). The scatter plot indicated that there was a significant overlap between the groups with and without postoperative post-neuraxial puncture-site pain. The fact that there is a significant overlap between the two clinical outcomes indicates that simple linear combinations of the baseline features may not be sufficient to separate them and hence the need to use advanced, potentially non-linear machine learning algorithms for risk prediction. In addition, the correlation heatmap (Figure 1B) showed that most clinical variables were rather independent from each other except for a biologically plausible positive correlation between the number of puncture attempts and the anesthesia operation time.

A comprehensive feature selection approach was used to find the most relevant features and avoid overfitting. Initial regularization of the coefficients using LASSO regression was applied (Figure 1C), and the penalty parameter was selected using cross-validation (Figure 1D). Next, a Random Forest algorithm was used to assess feature importance using Mean Decrease Gini metric (Figure 1E). Results indicated that BMI, age, intervention strategy and anesthesia operation time were the most prominent factors among the predictive model. Last, Recursive Feature Elimination (Figure 1F) suggested that the seven features that yielded the highest cross-validated accuracy are a subset of optimal features. As a result, these important characteristics were kept for the following training and testing of the machine learning models.

### 3.2 Evaluation of machine learning models

**Figure 2:**
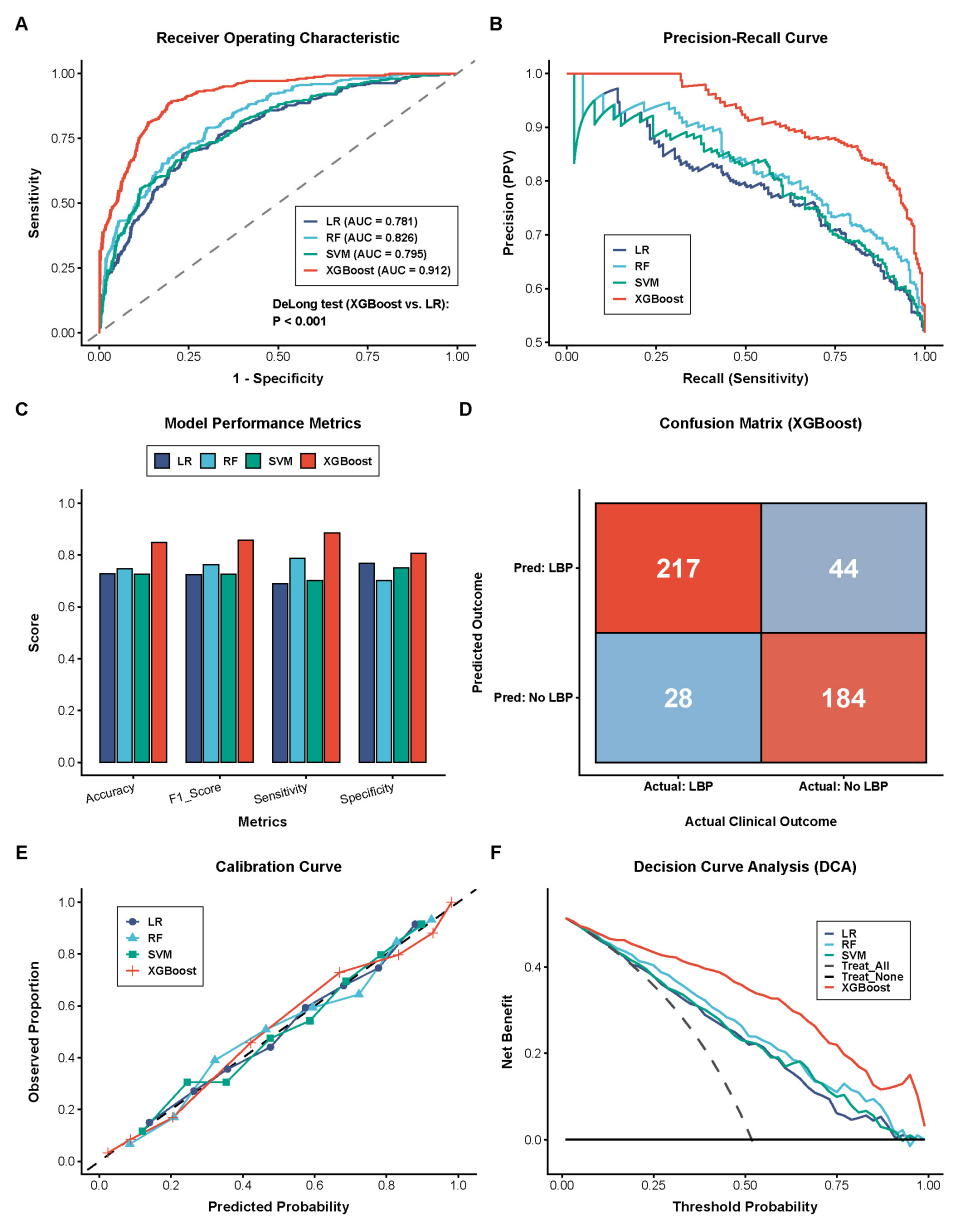
Evaluation of machine learning models. **(A)** Receiver Operating Characteristic (ROC) curves for the four models. **(B)** Precision-Recall (PR) curves. **(C)** Bar chart presenting the comparison of model performance metrics. **(D)** Confusion matrix of the via aggregated out-of-fold predictions across the entire cohort (*n* = 473). **(E)** Calibration curves assessing the agreement between predicted and observed probabilities. **(F)** Decision Curve Analysis (DCA) suggesting the potential net clinical benefit across different threshold probabilities.

Four different machine learning algorithms based on the optimal features identified in the previous step [Logistic Regression (LR), Random Forest (RF), Support Vector Machine (SVM), and XGBoost] were used to train and predict the occurrence of post-neuraxial puncture-site pain in the postoperative environment. The discriminative abilities of these models were first evaluated using Receiver Operating Characteristic (ROC) curves (Figure 2A). The XGBoost model showed the best discriminative performance (AUC = 0.912) followed by RF (AUC = 0.826), SVM (AUC = 0.795) and LR (AUC = 0.781). The DeLong test was used for the comparison of the AUCs of the two models of XGBoost and LR, and the test results showed that the difference was statistically significant (*P* < 0.001). This superiority indicates that the XGBoost model may have the ability to model the complex, non-linear interactions between clinical features, which is generally not possible with traditional linear models.

Due to the sensitivity of classification results to class distribution, Precision-Recall (PR) curves were also plotted to evaluate the precision of the models (Figure 2B). Overall, the XGBoost model outperformed the other models in terms of precision at different recall levels, demonstrating its ability to accurately classify positive cases. A comprehensive, quantitative summary of the predictive performance of the four baseline models derived from the aggregated out-of-fold predictions across the entire cross-validated cohort (*n* = 473) is detailed in Supplementary Table S1, including the AUC with 95% confidence intervals, sensitivity, specificity, accuracy, precision, recall, F1 score, Area Under the PR Curve (PR-AUC), Brier score, calibration slope, calibration intercept, and the optimal decision threshold for each model. Correspondingly, a visual comparison of key performance measures—including accuracy, F1 score, sensitivity, and specificity—is illustrated in Figure 2C. The XGBoost model had the highest performance on most measures. To gain a more detailed insight into the model's classification behavior, the confusion matrix for the XGBoost model, derived from the aggregated out-of-fold predictions across the entire cross-validated cohort (*n* = 473), is presented in Figure 2D. As would be true in a clinical predictive model, the matrix shows that the model was able to predict a large majority of actual clinical outcomes, but did leave a small number of false positives and false negatives.

Calibration curves were used to assess the accuracy of the predicted probabilities (Figure 2E). Overall, the curves, for all four models, appeared to be reasonably close to the ideal diagonal line, indicating that the predicted risk was reasonably close to the observed proportion. Some minor differences at the selected probability levels might be due to natural fluctuations in the clinical data. Finally, Decision Curve Analysis (DCA) was used to evaluate the possible clinical usefulness of the models (Figure 2F). The DCA indicated that across a wide range of threshold probabilities, utilizing the XGBoost model to guide clinical decision-making would provide a higher net benefit compared to the “treat-all” or “treat-none” strategies, as well as the other evaluated algorithms. Quantitatively, at a clinical threshold probability of 0.40, the net benefit of the XGBoost model was 0.352, which was substantially higher than that of the Logistic Regression model (0.224) and the “treat-all” strategy (−0.050), thereby supporting the superior clinical utility of the XGBoost algorithm.

### 3.3 Global SHAP explanations for the XGBoost model

**Figure 3:**
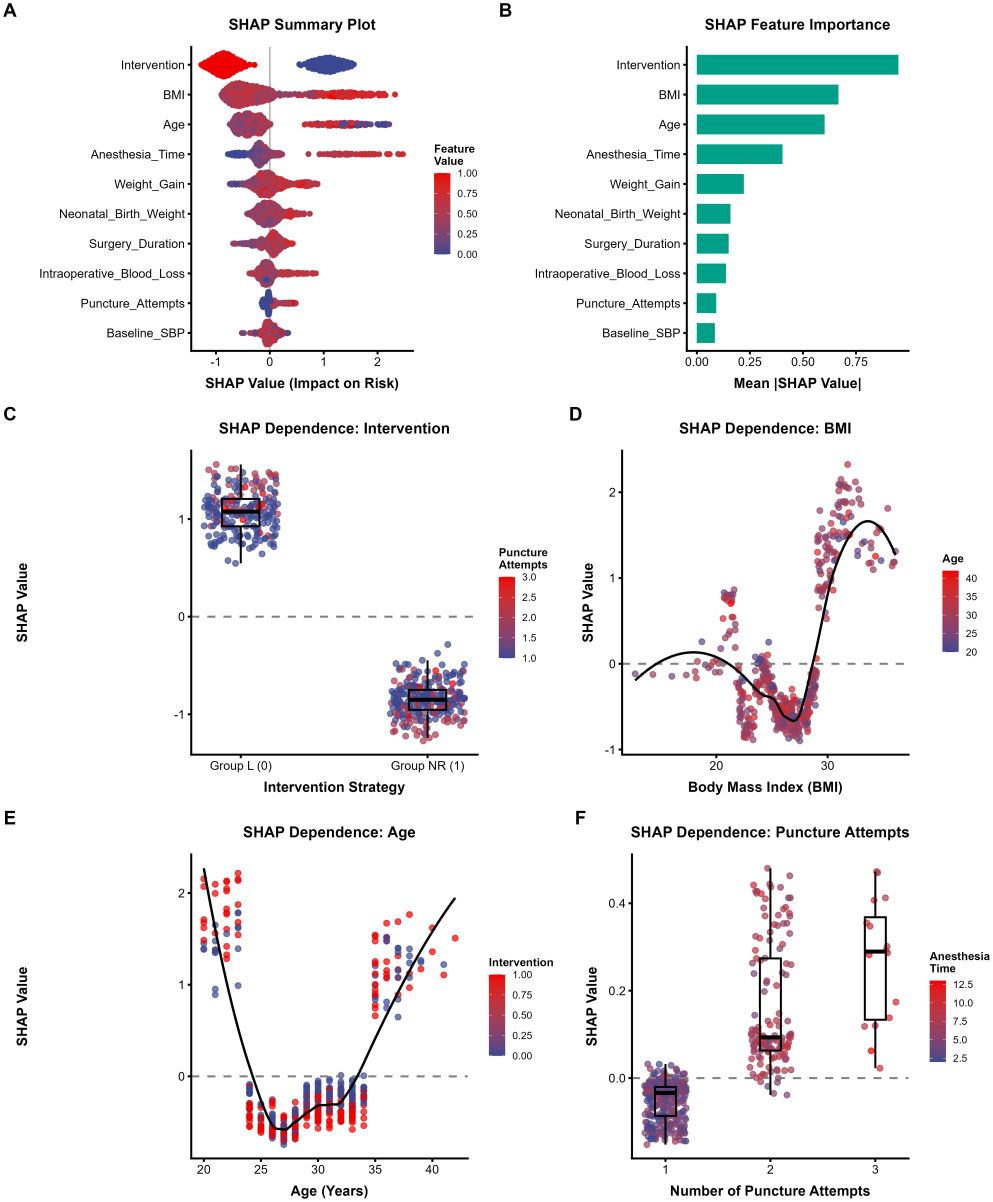
Global SHAP (SHapley Additive exPlanations) analysis for the XGBoost model. **(A)** SHAP summary plot displaying the distribution of feature impacts on the model output. **(B)** Mean absolute SHAP values indicating overall feature importance. **(C–F)** SHAP dependence plots illustrating the potential non-linear effects of intervention strategy **(C)**, body mass index **(D)**, age **(E)**, and number of puncture attempts **(F)** on the predicted risk.

In order to understand the decision-making process of the best XGBoost model, the SHapley Additive exPlanations (SHAP) framework was used to interpret the feature contributions to the model on a global scale. The SHAP summary plot (Figure 3A) shows the distribution of the impacts of individual features on the model's output. In this visualization, every point represents a single patient, with the color of each point reflecting the relative value of the feature and its horizontal coordinate representing the computed SHAP value. The intervention strategy emerged as the most prominent contributor to the overall model output. Specifically, high feature values of the intervention (represented by red dots, indicating the Nalbuphine group) were consistently associated with negative SHAP values, suggesting a potential statistical association with lower predicted pain probabilities. The SHAP feature importance bar plot (Figure 3B) further quantifies the overall magnitude of feature contributions, and indicates that the top three predictors are intervention strategy, body mass index and age.

SHAP dependence plots were generated to explore the relationship of each feature with the predicted risk. As seen in Figure 3C, the dependence plot of the intervention strategy, with overlaid boxplots, clearly showed a downward shift of the median SHAP value of patients who received the Nalbuphine intervention (Group NR) compared to the control group (Group L). This finding suggests that the intervention is linked with a decreased predicted probability of the outcome if there are some differences in the number of puncture attempts (shown by the color scale).

In addition, dependence plots of the continuous variables indicated non-linear relationships that may not be explained by linear models. The association of BMI with the SHAP value was clearly U-shaped as illustrated in the scatterplot smoothing (LOESS) curve (Figure 3D). Extreme deviations toward lower or higher BMI seemed to be associated with higher SHAP values, while the predicted risk seemed to reach a minimum around BMI of 24–27 kg/m^2^. TAge (Figure 3E) showed a similar non-linear relationship, with an increase in the predicted risk associated with advancing maternal age (e.g., >35 years). Finally, the dependence plot of the number of puncture attempts (Figure 3F) showed that median SHAP value was monotonically increasing with the number of puncture attempts. There was also an apparent multiplicative effect indicated by the color mapping of this plot: anesthesia operation time (red dots) tended to be concentrated in the top part of the boxplots, indicating an additive burden of risk. It is worth noting that the information this SHAP approach provides is one of statistical association that underpins the model's predictions and not causal association.

### 3.4 Local SHAP explanations and dynamic decision paths

**Figure 4:**
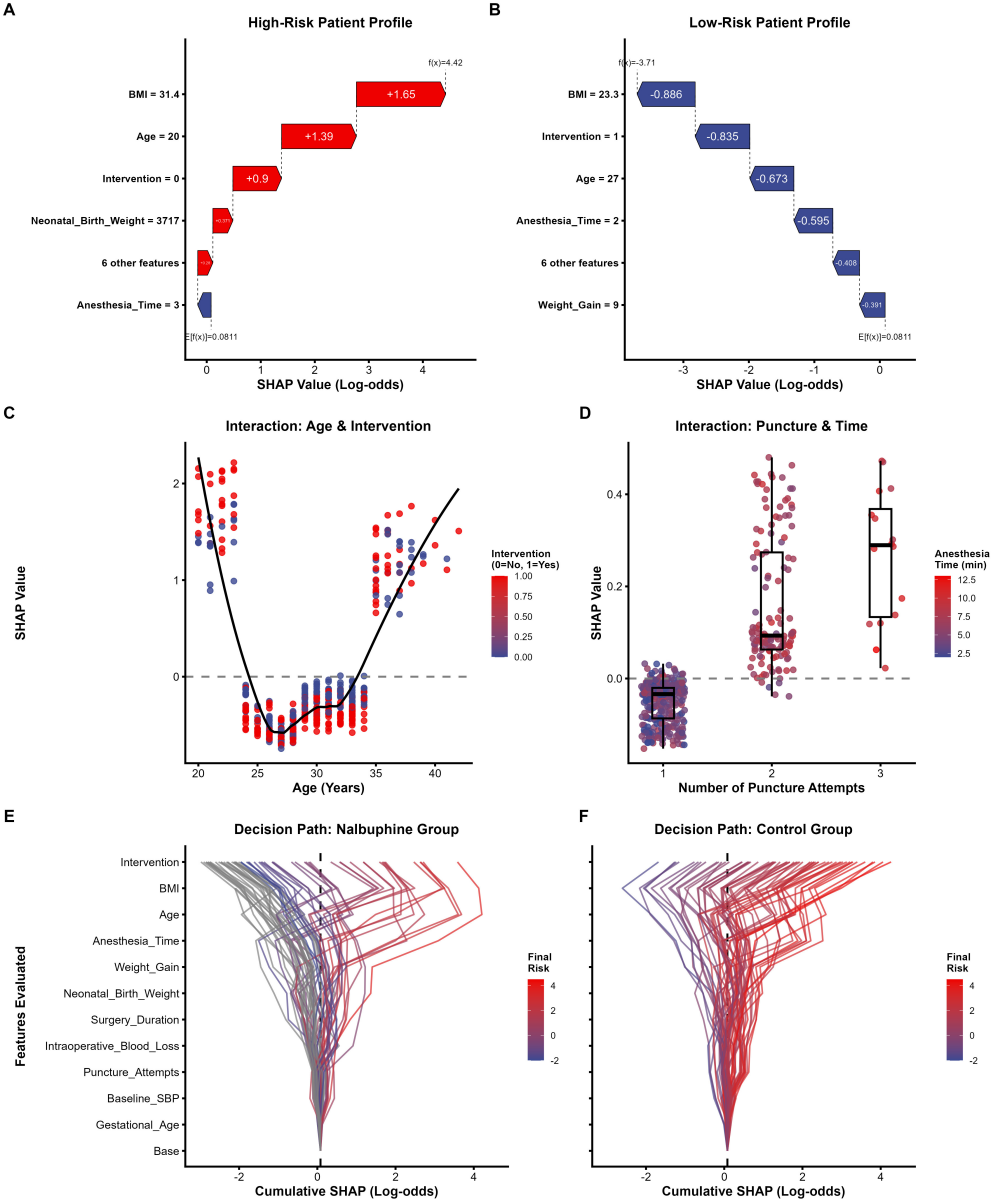
Local SHAP explanations and decision paths. **(A, B)** SHAP waterfall plots detailing the feature contributions for a representative high-risk **(A)** and low-risk **(B)** patient. **(C, D)** SHAP interaction plots exploring the potential interactive effects between age and intervention **(C)**, and between puncture attempts and anesthesia time **(D)**. **(E, F)** SHAP decision plots tracing the cumulative risk paths for a subset of patients in the Nalbuphine group **(E)** and the Control group **(F)**.

Global SHAP analysis provides an overview of how features contribute to the prediction, but assessing the contribution for each patient is important for precision medicine. Hence, local SHAP explanations were created using waterfall plots to explore the predictive logic of representative high-risk and low-risk parturients. In Figure 4A, a typical high risk patient profile is shown. Low or no Nalbuphine intervention (Intervention = 0) coupled with an extremely young age (20 years) and high body mass index (31.4 kg/m ^2^) synergized to act as strong risk factors and pushed her cumulative risk score well above baseline. In contrast, for a representative patient with low risk (Figure 4B), the use of epidural Nalbuphine (Intervention = 1) was the most important protective feature in the model (shown as a large negative SHAP value). Other minor variations in risk were offset in this patient profile by this intervention, a normal BMI, plus only one puncture attempt, and the final prediction score fell within the low-pain-risk zone.

SHAP interaction plots were also created to gain deeper insights into the effect of other clinical factors and how the intervention affects them. The interaction plot of age and intervention (Figure 4C) showed that the baseline risk was increased in general with age (e.g., >35 years); however, the use of Nalbuphine (red dots) seemed to reduce the risk elevation with age, bringing the SHAP values closer to or below the zero-effect line in some patients. Likewise, the combined effect of puncture attempts and anesthesia operation time (Figure 4D) revealed that the risk penalty from multiple punctures (e.g., 2 attempts) was not ubiquitous, but was apparent when used in combination with longer anesthesia operation time (dark red dots in the top 25% of the boxplot), implying an additive effect of procedural difficulty on postoperative pain risk.

Finally, SHAP decision plots were created for randomly selected groups of patients from Nalbuphine and Control groups and plotted on a common scale for objective comparison of risk accumulation across the entire set of features assessed. In the Control group (Figure 4F), the individual trajectories spread out quite a bit toward the right, showing an accumulation toward higher risk log-odds. The decision paths for the Nalbuphine group (Figure 4E), in contrast, displayed a leftward shift en masse. Such a unique visual divergence suggests that the Nalbuphine-Ropivacaine intervention is associated with a lower accumulation of risk features, thereby modifying the global picture of the postoperative pain trajectory of the study population.

### 3.5 Postoperative clinical outcomes and recovery profiles

**Figure 5:**
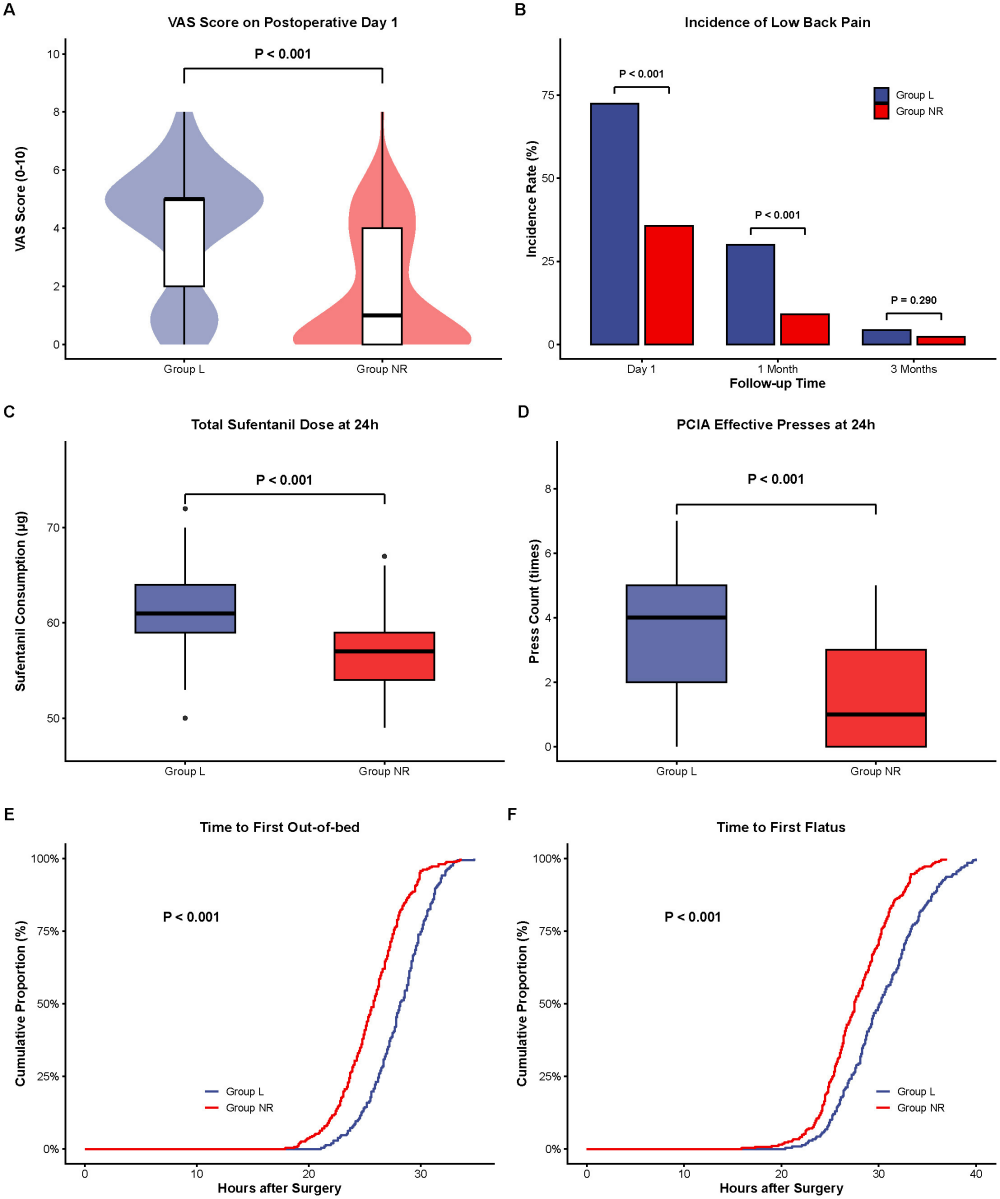
Comparison of postoperative clinical outcomes. **(A)** Violin plot with overlaid boxplot showing VAS scores on postoperative day 1. **(B)** Incidence rates of post-neuraxial puncture-site pain at different follow-up time points. **(C)** Boxplot of total sufentanil consumption at 24 h. **(D)** Boxplot of PCIA effective presses at 24 h. **(E, F)** Cumulative incidence curves illustrating the time to first out-of-bed **(E)** and time to first flatus **(F)**.

Traditional statistical analyses were performed to compare actual postoperative outcomes between the two groups to validate clinical implications suggested by the machine learning models. A violin plot in combination with a boxplot was used to evaluate the VAS scores on postoperative day 1 (Figure 5A). Results showed that pain intensity in Group NR was significantly lower than in Group L (*P* < 0.001), which was in line with the protection effect of Nalbuphine revealed by the SHAP analysis.

The numbers of post-neuraxial puncture-site pain cases at various follow-up time points were also measured longitudinally (Figure 5B). On postoperative day 1, the incidence rate in Group NR (35.7%) was significantly lower than that in Group L (72.5%, *P* < 0.001). In multivariable logistic regression adjusting for baseline confounding factors (including maternal age, body mass index, gestational age, weight gain during pregnancy, and the number of puncture attempts), the intervention remained independently associated with a lower risk of acute postoperative puncture-site pain on day 1 [adjusted odds ratio (aOR) = 0.210, 95% confidence interval (CI): 0.140–0.310, *P* < 0.001]. This lower incidence was maintained at the 1-month follow-up [unadjusted *P* < 0.001]. The incidence rates, however, decreased by the 3-month follow-up in both groups without a significant difference between them (*P* = 0.290). To rigorously control for confounding over time, longitudinal repeated-measures secondary outcomes (SAS, EPDS, and QoR-15 scores) were evaluated using Generalized Estimating Equations (GEE). The GEE-adjusted longitudinal trajectories, as visualized in Supplementary Figure S1, confirmed significant Group × Time interaction terms for QoR-15 (*P* = 0.004), SAS (*P* < 0.001), and EPDS (*P* = 0.002), indicating that the beneficial associations of local tract infiltration on maternal recovery and psychological trajectories remained robust after controlling for baseline covariates.

In terms of postoperative pain management, the exploratory association with reduced systemic opioid consumption was evaluated. The total consumption of Sufentanil at 24 h was significantly lower in Group NR than Group L (*P* < 0.001, Figure 5C); in multivariable linear regression controlling for baseline confounders, the intervention was independently associated with a significant decrease in 24-h sufentanil consumption [adjusted beta coefficient (β) = −4.300 μg, 95% CI: −5.100 to −3.500 μg, *P* < 0.001). Likewise, the number of effective Patient-Controlled Intravenous Analgesia (PCIA) presses at 24 h was significantly less in the Nalbuphine group (*P* < 0.001, Figure 5D) and remained independently associated with the intervention in adjusted analysis (β = −3.000 times, 95% CI: −3.300 to −2.700 times, *P* < 0.001). However, due to unmeasured variations in PCIA background settings and non-opioid co-analgesics across different protocol periods, these findings should be interpreted as exploratory associations rather than definitive intervention effects.

To systematically assess the safety profile of the intervention, maternal and neonatal adverse events were comprehensively recorded, as detailed in Supplementary Table S2. No serious complications, such as local puncture-site infection, epidural hematoma, or respiratory depression, were observed in either group. The incidences of common maternal side effects—including nausea and vomiting (12.1% in Group L vs. 9.0% in Group NR, *P* = 0.286), pruritus (7.7% in Group L vs. 5.3% in Group NR, *P* = 0.273), dizziness (4.8% in Group L vs. 4.1% in Group NR, *P* = 0.718), and urinary retention (5.8% in Group L vs. 5.3% in Group NR, *P* = 0.796)—showed no statistically significant differences between the groups. Neonatal outcomes, including 1-min and 5-min Apgar scores and delayed breastfeeding onset (7.2% in Group L vs. 6.0% in Group NR, *P* = 0.589), were also comparable, suggesting that localized nalbuphine-ropivacaine tract infiltration is associated with a favorable safety profile.

In addition, cumulative incidence curves were used for the analysis of indicators that were related to the Enhanced Recovery After Surgery (ERAS) pathways. Time to first out of bed mobilization (Figure 5E) and time to first flatus (Figure 5F) was significantly faster in Group NR (Log-rank test *P* < 0.001, both). Optimized pain management strategy may help patients to achieve these recovery milestones at early time points, as the red curves have steeper upward trajectories, representing a higher proportion of patients in the total group who are achieving the milestones at these earlier time points.

### 3.6 Subgroup analysis and longitudinal psychological assessments

**Figure 6:**
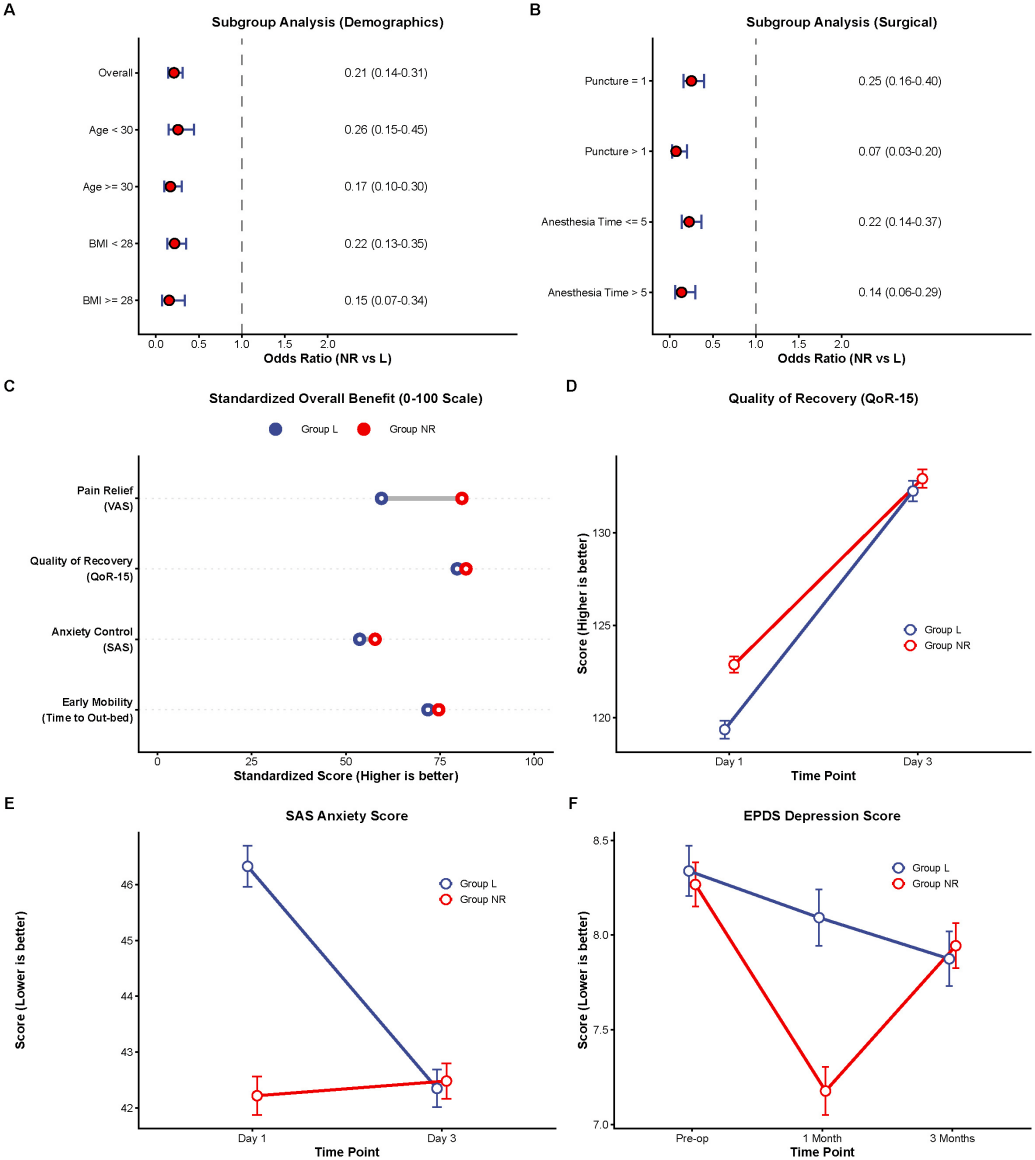
Subgroup analysis and longitudinal psychological assessments. **(A, B)** Forest plots presenting the odds ratios (ORs) for the intervention effect across demographic **(A)** and surgical **(B)** subgroups. **(C)** Dumbbell plot showing standardized overall benefit scores. **(D, F)** Longitudinal changes in Quality of Recovery-15 (QoR-15) score **(D)**, SAS anxiety score **(E)**, and EPDS depression score **(F)** over the study period. Error bars represent the standard error of the mean.

Subgroup analyses were conducted to evaluate the consistency of the protective effect from Nalbuphine-Ropivacaine intervention across different patient groups. The forest plots present the estimated odds ratios (ORs) and 95% confidence intervals (CIs) for the risk of postoperative post-neuraxial puncture-site pain according to demographic characteristics (Figure 6A) and surgical characteristics (Figure 6B). Quantitatively, within the demographic subgroups, the estimated OR was 0.260 (95% CI: 0.150–0.450) for age < 30 years (*n* = 221; 121 pain events) and 0.170 (95% CI: 0.100–0.300) for age ≥ 30 years (*n* = 252; 124 pain events), with no significant interaction detected (Pinteraction = 0.521). For the body mass index (BMI) subgroups, the OR was 0.220 (95% CI: 0.130–0.350) for BMI < 28 kg/m^2^ (*n* = 245; 129 pain events) and 0.150 (95% CI: 0.070–0.340) for BMI ≥ 28 kg/m^2^ (*n* = 228; 116 pain events; Pinteraction = 0.742). Within the surgical subgroups, the OR was 0.250 (95% CI: 0.160–0.400) for single puncture attempts (*n* = 328; 179 pain events) and 0.070 (95% CI: 0.030–0.200) for multiple puncture attempts (*n* = 145; 66 pain events; Pinteraction = 0.083). For anesthesia operation time, the OR was 0.220 (95% CI: 0.140–0.370) for duration ≤ 5 min (*n* = 270; 142 pain events) and 0.140 (95% CI: 0.060–0.290) for duration > 5 min (*n* = 203; 103 pain events; Pinteraction = 0.435). The ORs were consistently < 1.0 in all evaluated subgroups, suggesting that the exploratory protective association of the intervention remains stable regardless of baseline characteristics or procedural difficulty.

A dumbbell plot (Figure 6C) was used to visualize multiple clinical dimensions standardized on a 0–100 scale, to present an overview of the patients' postoperative recovery profiles. The greatest improvement in the net benefit of the Nalbuphine intervention was for the pain relief domain, with the longest gray area between Group NR and Group L. There were also relative advantages (rightward shift of red dots) in secondary domains such as early mobility and anxiety control, but these effect sizes were generally smaller than those for analgesia. To rigorously evaluate these longitudinal recovery trends over time while adjusting for covariates, repeated-measures QoR-15 scores were analyzed using GEE. The results, as illustrated in Supplementary Figure S1A, revealed a significant Group × Time interaction (*P* = 0.004), with GEE-adjusted marginal means showing a slight initial advantage for Group NR on Day 1 (122.9 vs. 119.4), followed by convergence to similar, high recovery scores by Day 3.

Longitudinal assessment of anxiety and depression was performed because it was believed that there may be a relationship between physical pain and psychological well being during the puerperium. In the GEE analysis of SAS anxiety scores (Supplementary Figure S1B), a highly significant Group × Time interaction was observed (*P* < 0.001), indicating that GEE-adjusted marginal anxiety scores were lower in Group NR on Day 1, with scores converging by Day 3. Similarly, GEE modeling of EPDS depression scores (Supplementary Figure S1C) demonstrated a significant Group × Time interaction at 1 month (*P* = 0.002). This indicates that the reduction in depression scores in Group NR was robust after adjusting for baseline covariates. By the 3-month follow-up, the GEE-adjusted EPDS scores converged again, suggesting that the psychological associations of the intervention are primarily observed during the early postpartum phase rather than exerting a permanent effect. This is a divergence that suggests a potential exploratory association where early intervention in puncture-site pain might be linked to a temporarily lower risk of early postpartum depressive symptoms, though this remains hypothesis-generating. EPDS scores were similar at 3-months follow-up indicating that the psychological impact of the intervention was more of the early postpartum period and did not have a lasting effect.

## 4 Discussion

Post-neuraxial puncture-site pain after cesarean section with neuraxial anesthesia is a common postoperative problem which can impair early maternal rehabilitation (18). In the current study, our findings indicate that local infiltration with nalbuphine-ropivacaine mixture can help decrease the occurrence of post-neuraxial puncture-site pain in the immediate postoperative period and decrease early postpartum depressive symptoms.

Traditional predictive analyses for obstetric anesthesia are linear models, which may not be able to reflect the complex clinical situation (19). The results showed that XGBoost algorithm had a better discriminative power than logistic regression. This enhancement is probably due to the algorithm's ability to recognize non-linear relationships. The predicted risk was significantly increased for extreme differences in BMI and increased maternal age as shown in the SHAP dependence plots. It is important to stress that the SHAP values show the statistical relationships that underlie the output of the model, and not cause and effect relationships (20). The increased incidence of the observed interaction between multiple puncture attempts and longer anesthesia time suggests that multiple localized tissue traumas may be an important risk factor and supports other studies reporting localized inflammatory reactions as a risk factor for needle-induced back pain (21).

Beyond predictive modeling, the clinical endpoints assessed in this study support the multidimensional benefits of the intervention. Nalbuphine is a kappa-opioid receptor agonist and previous studies have shown that it is an effective agent for producing regional analgesia and has an opioid-sparing effect (22). In the current study, we found less consumption of 24-h sufentanil in the intervention group, which is consistent with the previous studies (23). In addition, the longitudinal psychological assessments showed a well-defined time course. Improvement in the depression score at 1 month indicated early separation, which may buffer psychological distress in early postpartum. These scores then converge at 3 months suggesting that the favorable psychological associations of the intervention are mostly confined to the acute/early subacute recovery period. Clinically, this is plausible because there is a temporal overlap of when mechanical trauma occurs during pregnancy and when psychological issues are prevalent postpartum, and the psychological status of mothers is determined by a variety of multifactorial psychosocial factors other than a single component of regional anesthesia (24).

Several limitations of this study should be explicitly acknowledged. First, the observational and single-center nature of our design implies a lack of active randomization and assessor blinding during the intervention allocation, which introduces potential risks of selection bias, anesthesiologist preferences, and unmeasured confounders. Second, although we amended our criteria to exclude active pregnancy-related pain, pre-existing subclinical low back or pelvic girdle pain was not systematically adjusted for. Third, perioperative standardization was inherently limited by calendar-time confounding. We could not fully control or adjust for the specific protocol period, the attending anesthesiologist's preferences, detailed PCIA settings, intraoperative non-opioid co-analgesics, and postoperative mobilization protocols. Consequently, all observed differences in secondary outcomes (including opioid consumption, QoR-15, SAS, and EPDS scores) must be strictly interpreted as exploratory associations rather than causal intervention effects. Fourth, while the inclusion of the intervention strategy as a predictor limits the model's utility as a pure pre-operative, static screening tool, it is critical to recognize that the SHAP-based interpretations in this study only reflect model-based statistical associations, not counterfactual causal effects. The model cannot be used to simulate or estimate the true individual benefit of the intervention for a specific patient, as the intervention variable inherently captures unmeasured confounding related to clinician preference and protocol periods. Nonetheless, future studies should focus on validating a baseline-only risk-prediction model in untreated patients to establish a pure pre-operative screening tool before clinical decisions are made. Fifth, while our 5-fold cross-validation workflow ensured robust internal evaluation without data leakage, the absence of an external, independent hold-out test cohort may constrain the generalizability of the reported performance metrics. Future external validation in independent obstetric cohorts is essential before clinical deployment. Lastly, the systematic monitoring of adverse events was restricted to documented medical records, which may have led to the underreporting of subtle safety signals.

## 5 Conclusion

In summary, based on this study, the exploratory findings suggest that local application of nalbuphine with ropivacaine exhibits a potential statistical association with a reduced need for postoperative opioids and the incidence of acute postoperative post-neuraxial puncture-site pain after cesarean section. Non-linear relationships and feature interactions, such as BMI, age and procedural difficulty, were well captured by our machine learning method, especially the XGBoost algorithm, showing strong ability to identify the risk of postoperative pain. Moreover, alleviation of physical pain suggested an exploratory link with a potential reduction in early postpartum depressive symptoms, thus adding to improved early recovery. However, these exploratory associations require further confirmation in properly designed randomized controlled interventional studies and externally validated predictive models.

## Data Availability

The raw data supporting the conclusions of this article will be made available by the authors, without undue reservation.
