# Supplementary material for: Interpreting the risk of postoperative post-neuraxial puncture-site pain after cesarean section: a machine learning approach evaluating nalbuphine infiltration along the epidural puncture tract in a prospective observational cohort
Source: Front Med (Lausanne). 2026 Jul 29;13:1908970. doi: 10.3389/fmed.2026.1908970 (PMC13461617; doi:10.3389/fmed.2026.1908970)
Supplement: Supplementary file 1 [file Table_1.DOCX]

Figure S1. Generalized Estimating Equations (GEE)-adjusted longitudinal trajectories of postoperative recovery and psychological parameters.

(A) Estimated marginal means of Quality of Recovery-15 (QoR-15) scores on postoperative days 1 and 3.

(B) Estimated marginal means of Self-Rating Anxiety Scale (SAS) scores on postoperative days 1 and 3.

(C) Estimated marginal means of Edinburgh Postnatal Depression Scale (EPDS) scores at pre-operative, 1-month, and 3-month postpartum follow-ups.

All trajectories and error bars represent GEE-derived marginal means and their corresponding 95% confidence intervals, statistically adjusted for baseline confounders including maternal age, body mass index, and the number of puncture attempts. The annotated P-values represent the statistical significance of the Group × Time interaction terms, suggesting potentially divergent recovery and psychological trajectories between Group NR (red) and Group L (blue) over time.
